# Developmental and Reproductive Outcomes in Male Rats Exposed to Triclosan: Two-Generation Study

**DOI:** 10.3389/fendo.2021.738980

**Published:** 2021-10-13

**Authors:** Bruno Garcia Montagnini, Simone Forcato, Karine Vandressa Pernoncine, Mariana Cunha Monteiro, Marina Rangel Ferro Pereira, Nathalia Orlandini Costa, Estefânia Gastadello Moreira, Janete Aparecida Anselmo-Franci, Daniela Cristina Ceccatto Gerardin

**Affiliations:** ^1^ Laboratory of Pharmacology of Reproduction, Biological Sciences Center, Department of Physiological Sciences, State University of Londrina, Londrina, Brazil; ^2^ Department of Morphology, Stomatology and Physiology, Dental School of Ribeirão Preto, University of São Paulo, São Paulo, Brazil

**Keywords:** endocrine disruptors, reproduction, behavior, fertility, sperm

## Abstract

Triclosan (TCS) is a phenolic compound with broad-spectrum antimicrobial action that has been incorporated into a variety of personal care products and other industry segments such as toys, textiles, and plastics. Due to its widespread use, TCS and its derivatives have been detected in several environmental compartments, with potential bioaccumulation and persistence. Indeed, some studies have demonstrated that TCS may act as a potential endocrine disruptor for the reproductive system. In the current study, we are reporting on the results obtained for male rats after a two-generation reproduction toxicity study conducted with TCS. Female and male Wistar rats were treated daily by gavage with TCS at doses of 0.8, 2.4, and 8.0 mg/kg/day or corn oil (control group) over 10 weeks (F0) and over 14 weeks (F1) before mating and then throughout mating, until weaning F2 generations, respectively. TCS exposure decreased sperm viability and motility of F1 rats at the dose of 2.4 mg/kg. The effects of TCS on sperm quality may be related to the exposure window, which includes the programming of reproductive cells that occurs during fetal/neonatal development.

## 1 Introduction

Endocrine disruptors are referred to as environmental contaminants, described as an exogenous substance or mixture that alters function(s) of the endocrine system with adverse health effects in an intact organism, its progeny, or (sub)populations (1, 2). Several studies have shown a connection between phenolic environmental pollutants and endocrine-disrupting effects on the reproductive system (3–5).

Triclosan (TCS) is a trichlorinated phenoxyphenol with broad-spectrum antimicrobial action that has been incorporated into a variety of consumer products including soaps, hand sanitizers, toothpaste, mouthwash, and lotions, as well as being used in other consumer products such as toys, the textile industry, and plastics (6–8). The structural similarity of TCS to thyroid hormones and other organic environmental pollutants, such as polychlorinated-biphenyls, polybrominated diphenyl ethers, and bisphenol-A, may predict possible endocrine disrupting effects of TCS on reproductive and thyroid endpoints (9–12).

The worldwide annual consumption of TCS in 2015 was approximately 4,600 tons (13). Because of its widespread use, TCS and its derivatives have been detected in the effluent of wastewater treatment plants across the globe, representing one of the most commonly detected contaminants in solid and water environmental compartments (14, 15). Indeed, the aromatic nature of TCS and its high chlorine content make it resistant to degradation and persistent in the environment (16). In humans, TCS has been detected in urine samples from pregnant women (17) and in maternal plasma and breastmilk (18). It is also known that TCS crosses the placenta (19).

The continuous detection of TCS including its degradation products in the environment (20–22) raises concerns about the safety, effectiveness, and regulation of TCS usage. According to a review by Giuliano and Rybak (2015) (23), data from laboratory-based studies were unable to demonstrate considerable efficacy of TCS soap over non-antimicrobial soap. In 2016, Food and Drug Administration (FDA) established a ban on TCS and other antibacterial compounds used in consumer soaps and other washing products in the United States (U. S.) (24).

Some experimental evidence has demonstrated that TCS may act as a potential endocrine disruptor for the reproductive system. *In vitro*, TCS exposure suppressed testosterone production in two Leydig cell-based assays (25, 26). In humans, there is evidence that exposure to TCS is associated with reduced sperm concentration, sperm count, the number of forward-moving sperm, and an increased percentage of sperm with abnormal morphology (3, 27). In immature Wistar rats (23 days old), oral treatment (200 mg/kg) for 31 days decreased serum testosterone (28), while in adult Wistar rats (10 weeks old), decreased serum levels of androgens followed by reduced daily sperm production were observed after 60 days of oral treatment with TCS (20 mg/kg) (26). In Holtzman rats, maternal exposure to TCS, by subcutaneous injections from day 6 of gestation until day 21 of lactation, induced dose-dependent effects in male pups, decreasing plasma testosterone levels; sperm motility, and count in cauda of the epididymis at the doses 4, 40, and 150 mg/kg. The highest dose also induced a significant decrease in the number of seminiferous tubules, and weight of testis and seminal vesicle of the F1 male offspring (29). Moreover, evidence shows that TCS exposure decreased rodent and human fetal body weight and viability by inhibition of plasma estrogen sulfotransferase, which modulates estrogen activity (30). Regarding the neurodevelopmental hazard potential of TCS, there is one study conducted on zebrafish embryos, showing TCS exposure at concentrations between 2 to 2.8 µM delayed development of secondary motor neurons (31).

In this sense, considering (1) the widespread use of TCS and the risks involving its environmental exposure over the decades; (2) the potential TCS toxicity to the reproductive system and organism development; and (3) that the exposure to endocrine disruptors during early development may cause a growing set of diseases (32, 33), which is correlated with the concept of the Developmental Origins of Health and Disease (DOHaD), the present study aimed to evaluate the potential effects of TCS applying a two-generation reproduction toxicity study (34), which is designed to provide general information concerning the effects of a test substance on the integrity and performance of reproductive systems, including gonadal function, mating behavior, conception, gestation, parturition, lactation, and weaning, and the growth and development of the offspring. This work reports the outcome of F1 and F2 generations of male Wistar rats exposed to TCS.

## 2 Material and Methods

### 2.1 Study Design

The two-generation reproduction toxicity study was based on the OECD Guideline for Testing of Chemicals 416 (34), and the developmental neurotoxicity study (F1 and F2 generations) was based on the OECD guideline 426 (35).

### 2.2 Drug and Dosage

TCS was obtained from Vivimed Labs Limited (Habsiguda Hyderabad, India) (CAS no. 3380-34-5, 99.38% pure) and dissolved in corn oil (vehicle), before being administered orally, by gavage, in a volume of 2.5 ml/kg.

The description of the dose obtention was published previously in Pernoncini et al. (36). According to the U. S. Environmental Protection Agency (8), the acceptable daily intake of TCS is up to 0.3 mg/kg, for humans. The allocation factor adopted for TCS exposure in drinking water is 0.2 (37, 38), which corresponds to 20% of the acceptable daily intake value. In this sense, starting from the acceptable daily intake for humans, excluding the allocation factor, the BW3/4 dosimetric adjustment was applied considering the rodent metabolism (39). The weight of a human of 70 kg was considered and a rodent of 250 g to obtain the value of 0.8 mg/kg as the correspondent acceptable daily intake value for rats. The doses of 2.4 mg/kg and 8.0 mg/kg were derived with the application of security factors of 3 and 10, respectively, assuming the intraspecies variations.

### 2.3 Animals

Seventy-five-day-old male and female Wistar rats from the colony of the State University of Londrina were maintained in the animal facilities of the Department of Physiological Sciences in a controlled environment at a temperature of 21 ± 2°C, 12 h light/dark cycle (lights on at 6:00 a.m.), with free access to regular lab chow (Nuvilab™, Quimtia SA, Brazil) and water. Animals were housed in collective polypropylene cages (29 × 18 × 13 cm) with wood shavings as bedding and were mated after 1 week of acclimatization. Two females were mated with one male to obtain the F0 generation. The day of birth was considered postnatal day (PND) 0. All animal procedures were approved by the State University of Londrina Ethics Committee for Animal Research (CEUA/UEL: 283.2015.27).

### 2.4 Two-Generation Reproduction Toxicity Study

Male and female Wistar rats were used as the F0 generation (15–17 rats/sex/group). On PND 49, animals (both sexes) were randomly allocated, stratified by weight, to one out of three treatment groups to receive oral TCS doses of 0.8, 2.4, and 8.0 mg/kg, in addition to the control (CTR) group, treated with vehicle alone. Animals were treated once a day (11:00 a.m.–1:00 p.m.) for 70 days in order to cover at least one complete spermatogenic cycle and several complete cycles of oogenesis. At PND 120, animals were mated (as described below). Treatment continued throughout mating as well as pregnancy and lactation for the F0 females (i.e., until weaning of F1 pups), while in F0 males it continued for 20 days after mating in order to replace the spermatic stock.

For mating, F0 animals were cohabitated overnight daily (one male and one not sibling same-dose female per cage) until a positive pregnancy diagnosis. Females with sperm observed in the vaginal smear were considered as day 0 of the presumed gestational day (GD) and housed individually. Pregnant females were allowed to deliver their pups naturally. The day on which parturition was completed until 1:00 p.m. was designated as lactation day (LD) 0 for dams and PND 0 for pups. All live pups (F1 generation) were identified by skin tattoo on PND 1. Litters were culled on PND 4 to 8 pups, keeping four males and four females whenever possible and identified by ear marking at weaning (PND 21). Three male pups were used for the F1 analysis: one to evaluate the sexual development, physical development, and neuromotor development; other for motor activity; and another one for mating and sexual parameters.

The third male and one female (not sibling) were selected to parent the F2 generation. They were gavaged, following the same treatment protocol already described, from PND 22 (24 h after weaning) up to weaning of the F2 offspring, in this way, covering the mating period, which occurred around PND120, gestation and lactation. The F2 pups were exposed to TCS through F0 and F1 generation, placenta, and lactation and were evaluated only until weaning. The animal health status and clinical signs (e.g., lacrimation, piloerection, unusual respiratory pattern, and tremors) were checked daily.

**Figure 1:**
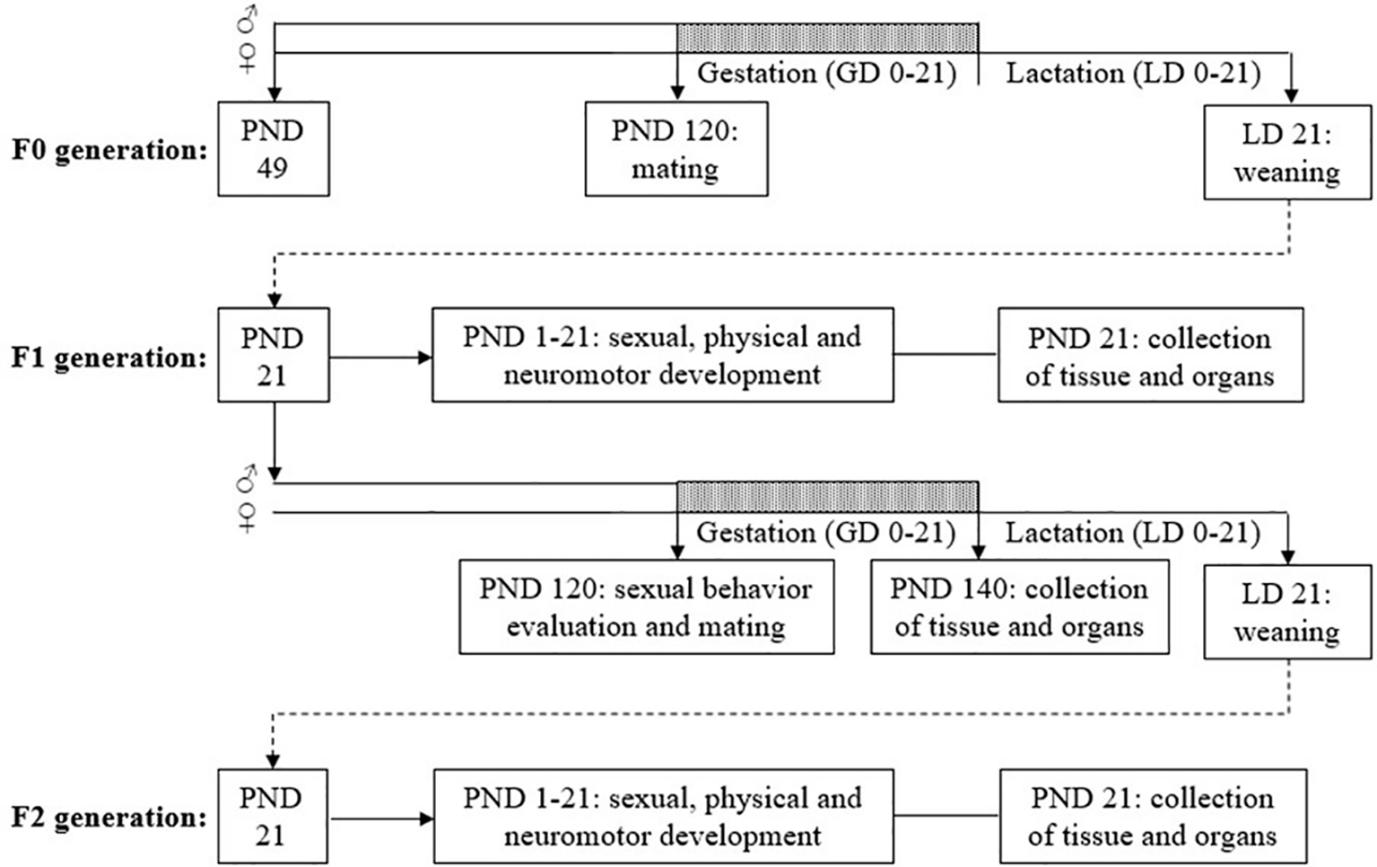
Diagram of the experimental design. GD, gestational day; LD, lactational day; PND, postnatal day.

The females from F0, F1 and F2 were used in another study to determine the reproductive toxicity to TCS exposure, and the results have already been published in Montagnini et al. (40). The data from F0 males are described in Pernoncini et al. (36). This study is reporting on the F1 and F2 male results. The diagram of the experimental protocol is shown in **Figure 1**.

#### 2.4.1 Body Weight and Food Consumption During the Pre-Mating Period

Body weight was measured every 3 days, from the beginning of the dosing period (F0 = PND 49; F1 = PND 22) to the day before mating (PND 120), considered as the pre-mating period (CTR n = 17; TCS 0.8 n = 15; TCS 2.4 n = 15; TCS 8.0 n = 15). Food consumption was obtained weekly during this period.

#### 2.4.2 Sexual Behavior Evaluation

All behavioral assessments were performed in adult rats (beginning on PND 120) (CTR n = 10; TCS 0.8 n = 8; TCS 2.4 n = 8; TCS 8.0 n = 8) during the dark phase of a reversed light/dark cycle, under dim red light. The animals were allowed 15 days of adaptation to the reversed light/dark cycle before the beginning of evaluation. The observations always started 2/3 h after the onset of darkness and were recorded by a video camera, linked to a monitor in an adjacent room.

##### 2.4.2.1 Copulatory Behavior

For the evaluation of copulatory behavior, each male was placed into a Plexiglass cage with dimensions 20 × 40 × 50 cm (height × width × length), and after 5 min, a non-treated female in natural estrous was introduced into the cage. For 30 min, the latencies and number of intromissions and ejaculations were observed as described previously in Gerardin et al. (41). If a male did not mount within 10 min, the evaluation was interrupted and repeated another day with another female. If the male failed again in the second evaluation, it was considered sexually inactive (42).

##### 2.4.2.2 Sexual Incentive Motivation

The same animals evaluated for copulatory behavior were submitted to the sexual incentive motivation test. In this test, a rectangular arena with dimensions 50 × 50 × 100 cm (height × width × length) was used, with two openings that lead to two small arenas of 25 cm^2^. The small arenas are diagonally opposed to each other, and the communication with the main arena is closed with a wire mesh. For the test, an estrous female (female zone) was placed in one of the small arenas and a sexually active male (male zone) was placed in the other arena. The floor of the main arena had two 25 cm^2^ divisions (zones) in front of each small arena opening, named female and male incentive zones, respectively. The experimental male was placed in the center of the main arena and observed for 20 min. The number of visits and the total time spent visiting each zone were quantified, and a preference score was calculated as (time spent in the female zone/total time spent in both incentive zones) × 100 (43).

#### 2.4.3 Collection of Organs

The F1 males used for sexual behavior evaluation were euthanized by decapitation 20 days after mating (CTR n = 10; TCS 0.8 n = 8; TCS 2.4 n = 8; TCS 8.0 n = 8). Blood was collected for testosterone quantification. The testis (pair), vas deferens, epididymis (left), ventral prostate, liver, kidneys (pair), adrenal glands, and seminal vesicle (pair) (without the coagulating gland and full of secretion) were removed and their weights determined. The right testis and epididymis were frozen at −80°C for sperm counting, and the left testis was collected for histomorphometric analysis. The right vas deferens was used for the analysis of motility, viability, and sperm count and the left for sperm morphology.

#### 2.4.4 Plasma Testosterone Quantification

Blood samples were collected from the abdominal aorta into syringes containing heparin, then centrifuged (2,500 rpm for 20 min at 4°C), and the plasma frozen until assayed. Plasma testosterone quantification was measured by chemiluminescence microparticle immunoassay (ARCHITECT™, 2nd Generation Testosterone, Abbott Laboratories, IL, USA) according to the manufacturer’s instructions, with an intra-assay coefficient of variation and minimum sensitivity of the assay of 4.6% and 0.15 nmol/L, respectively.

#### 2.4.5 Sperm Parameters

##### 2.4.5.1 Sperm Motility, Viability, Sperm Count in the Vas Deferens, and Morphology

Sperm motility analysis was performed according to Perobelli et al. (44) and Favareto et al. (45). Sperm was obtained from the right vas deferens and diluted in 1 ml of GV HEPES medium (Ingamed, Maringá, Brazil) prewarmed at 34°C. A 10 μl aliquot was placed in a Makler chamber (Sefi-Medical, Haifa, Israel) and analyzed under a phase-contrast microscope at 100× magnification. One hundred sperms were evaluated per animal (CTR n = 10; TCS 0.8 n = 8; TCS 2.4 n = 8; TCS 8.0 n = 8) and were classified as motile or immotile.

For sperm viability, the one-step eosin–nigrosin staining technique was used (46). A total of 50 μl of sperm in GV HEPES medium (Ingamed, Maringá, Brazil) was mixed with eosin–nigrosin and directly examined, and 100 sperms per animal were evaluated and classified as viable or non-viable.

Sperm count in the vas deferens (10^6^/ml) was performed in a Makler chamber (Sefi-Medical, Haifa, Israel). Sperm was counted in 10 different squares chosen at random, in four different fields, and the average was obtained (47).

The evaluation of sperm morphology was performed according to Fernandes et al. (48). Sperm was recovered from the left vas deferens by flushing with 1 ml of formol-saline (10%), and smears were prepared on histological slides that were left to dry for 90 min. In total, 200 sperms were analyzed per animal in a phase-contrast microscope (400× magnification) (49). Morphological abnormalities were classified into two general categories: head morphology (without characteristic curvature or isolated form, i.e., no tail attached) and tail morphology (broken or isolated i.e., no head attached) (50).

##### 2.4.5.2 Daily Sperm Production Per Testis, Sperm Number, and Transit Time in the Epididymis

The left testis was decapsulated, and the caput/corpus and cauda segments from the epididymis were separated. Homogenization-resistant testicular spermatids (stage 19 of spermiogenesis) and sperm in the caput/corpus epididymis and cauda epididymis were assessed as previously described by Robb et al. (51) with adaptations of Fernandes et al. (48). Mature spermatids were counted in a Neubauer chamber. To calculate daily sperm production (DSP), the number of spermatids at stage 19 was divided by 6.1, which is the number of days in one seminiferous cycle when these spermatids are present in the seminiferous epithelium. Sperm transit time through the epididymis was determined by dividing the number of sperm in each segment by the DSP (CTR n = 10; TCS 0.8 n = 8; TCS 2.4 n = 8; TCS 8.0 n = 8).

#### 2.4.6 Testicular Histomorphometry

The right testis (CTR n = 10; TCS 0.8 n = 8; TCS 2.4 n = 8; TCS 8.0 n = 8) was promptly dissected, weighed, and fixed by immersion in Bouin’s solution for 24 h before being stored in ethanol at 70°. The testis was cut into tissue fragments and routinely processed for embedding in Paraplast™ blocks. Six semi-serials sections of 7 µm separated by a distance of 50 μm per section were obtained from each animal and stained with hematoxylin-eosin. The diameter of the seminiferous tubules was measured at 100× magnification using an ocular micrometer. Fifteen cross-sections of tubule profiles that were either round or nearly round were chosen randomly and measured for each animal. The volume densities of testicular tissue components were determined by light microscopy using a 100-intersection grid placed in the ocular of the light microscope. A total of 10 (1,000 points) randomly chosen fields were scored for each animal at 400× magnification. The volume of each component of the testis was determined as the product of the volume density and testis volume. For subsequent stereological calculations, the specific gravity of testis tissue was 1.0 (52). To obtain a more precise measure of testis volume, the testis capsule (∼6.5%) was excluded from the testis weight. The total length (in meters) of seminiferous tubules (LST) was estimated by the tubule seminiferous volume (TSV) in the testis and the average area of tubules obtained from each animal (πR^2^; R = tubular diameter/2, according to the formula: LST = TSV/πR^2^) (53, 54).

### 2.5 Offspring Evaluation (F1 and F2 Males)

All offspring of F1 (CTR n = 17; TCS 0.8 n = 14; TCS 2.4 n = 14; TCS 8.0 n = 15) and F2 (CTR n = 14; TCS 0.8 n = 12; TCS 2.4 n = 12; TCS 8.0 n = 11) generations were examined as soon as possible on the day of birth (PND 0) to determine the number of liveborn and stillborn per litter; the pups were sexed based on the anus to genital tubercle distance. Pups were weighed on PNDs 1, 4, 7, 14, and 21. Pup health status and clinical signs were checked at least once daily throughout lactation.

#### 2.5.1 Sexual Development

Sexual development data were obtained from one randomly selected male of each litter from F1 (CTR n = 17; TCS 0.8 n = 14; TCS 2.4 n = 14; TCS 8.0 n = 15) and F2 (CTR n = 14; TCS 0.8 n = 12; TCS 2.4 n = 12; TCS 8.0 n = 11) generations. The anogenital distance (AGD, distance from the anus to the genital tubercle) was obtained through a Vernier caliper on PNDs 1, 4, and 21. Relative AGD was normalized through its division by the cube root of body weight (55). Moreover, the male pups were examined for the presence of nipples/areolas on PND 12.

The age at complete preputial separation was recorded to investigate puberty onset and was recorded only from F1 males selected for mating.

#### 2.5.2 Physical Development

The following physical developmental parameters were observed: pinna detachment (unfolding of external ear) (from PND 2), incisor teeth eruption (from PND 6), and eye opening (from PND 10) (F1 generation: CTR n = 17; TCS 0.8 n = 14; TCS 2.4 n = 14; TCS 8.0 n = 15; F2 generation: CTR n = 14; TCS 0.8 n = 12; TCS 2.4 n = 12; TCS 8.0 n = 11). Age at eye opening and ear detachment was recorded when both eyes and ears were completely opened. The incisor eruption was examined daily to determine the first day of the bilateral appearance of the upper incisors.

The same pup selected for sexual and physical development evaluations was used for all neuromotor behavior assessments (F1 generation: CTR n = 17; TCS 0.8 n = 14; TCS 2.4 n = 14; TCS 8.0 n = 15; F2 generation: CTR n = 14; TCS 0.8 n = 12; TCS 2.4 n = 12; TCS 8.0 n = 11). The dependent variable analyzed for each test was the day of first achieving either maturity of the reflex or the conditions listed below.

#### 2.5.3 Neurobehavioral Tests

The neurobehavioral assessments were evaluated following the OECD/OCDE Guideline 426 for Developmental Neurotoxicity Study (35) by a trained observer masked to experimental groups. The protocol of these developmental tests is used to assess the impact of early prenatal and/or postnatal insults such as xenobiotic exposure (56).

Righting reflex: Starting on PND 5, the pup was placed on its back on a flat surface and allowed to right itself. Time to regain normal position was recorded. This reflex was completed if the pup performed this response within 10 s.

Negative geotaxis: Starting on PND 5, each rat was placed on an inclined wire mesh ramp (angle of inclination from the base: 45°) with the head facing down. The reflex was acquired when pups performed a 180° body rotation and when they could climb up within 30 s.

The righting reflex is a test used to investigate the reflection of sensorimotor coordination, while the negative geotaxis reflex evaluates the cerebellar integration. In rodents, these reflexes arise on average at PND 5 and at PND 7, respectively (56).

Motor activity: Motor activity behavior of offspring on PNDs 13, 17, and 21 was evaluated in an open-field arena in F1 (CTR n = 17; TCS 0.8 n = 14; TCS 2.4 n = 14; TCS 8.0 n = 15) and F2 pups (CTR n = 14; TCS 0.8 n = 12; TCS 2.4 n = 12; TCS 8.0 n = 11). The apparatus consists of a circular wooden surface with a diameter of 39 cm, surrounded by a wooden wall 28 cm in height. The surface is painted white and divided into 19 similar parts by concentric circles and straight segments. Each animal was placed in the center of the arena and its locomotion evaluated (number of spaces invaded by the animal with all four legs) during a 10-min session (57). After the removal of each animal, the floor was carefully cleaned with a cloth embedded with a 5% ethanol solution. The test was carried out between 09:00 a.m. and 03:00 p.m. in a quiet room.

### 2.6 Statistical Analysis

An exploratory analysis was conducted to evaluate the normal distribution (Shapiro-Wilk test) and homogeneity of variance (Levene’s test) of each variable. In the absence of normal distribution and/or homogeneity of variance, variables were transformed in order to achieve the criteria for parametric analysis. Variables that presented normal distribution and homogeneity of variance were analyzed by ANOVA complemented with the Bonferroni *post-hoc* test, with data being presented as mean ± standard error of the mean (SEM). Conversely, for other variables the Kruskal-Wallis complemented with Dunn’s test was performed with the presentation of data as median (1st and 3rd quartiles). Analysis of covariance (ANCOVA) was used to detect the effect of the treatment on organ weight using the final body weight as the covariate. However, since the ANCOVA and ANOVA provided similar results, the ANOVA results are reported. For pup body weight, relative AGD, and motor activity, a repeated measures ANOVA (RMANOVA) was applied with day as the within-subject factor and treatment as the between-subject factors. The Chi-Square test was used to examine differences in categorical variables (nipple retention, frequency of ejaculations, and animals that displayed copulatory behavior). Differences were considered significant if p < 0.05. All statistical analyses were performed using SPSS (IBM, SPSS Statistics v19).

## 3 Results

### 3.1 Parameters Analyzed in Adult Rats (F1 Generation)

#### 3.1.1 Clinical Observations, Body Weight, and Food Consumption (Premating Period)

No mortality, morbidity, or general signs of toxicity such as changes in behavior (agitation, lethargy, and hyperactivity), neurological changes (convulsions, tremors, muscle rigidity, and hyperreflexia), or autonomic signs (lacrimation, piloerection, and unusual respiratory patterns) were observed during the treatment period.

Body weight gain was calculated from the beginning of the dosing period (PND 22) to the day before mating (PND 120). As described by ANOVA, the chronic administration of TCS did not affect body weight gain (g) during the pre-mating period of F1 rats (CTR group: 403.63 ± 13.07, n = 17; TCS 0.8 group: 418.63 ± 13.63, n = 15; TCS 2.4 group: 417.42 ± 17.77, n = 15; TCS 8.0 group: 415.66 ± 13.58, n = 15) [F_(3,61)_ = 0.239, p = 0.869]. No treatment effect of TCS was observed in the mean feed consumption of F0 and F1 generation groups during these periods (data not shown).

#### 3.1.2 Sexual Behavior Evaluation

The results of sexual behavior (copulatory behavior and sexual incentive motivation) of male rats of the F1 generation at PND 120 are shown in **Table 1**. No statistical differences were detected in the parameters of copulatory or sexual incentive motivation parameters (p > 0.05). There were no significant differences between the percentage of animals that displayed copulatory behavior and the frequencies of ejaculations of the TCS-treated groups compared to the CTR group (Chi-Square test, p > 0.05) (data not shown).

**Table 1:** Sexual behavior of male rats of F1 generation at PND 120.

Copulatory behavior	CTR	TCS 0.8	TCS 2.4	TCS 8.0
Latency to the first intromission (s)	129.00 (89.75–246.50) [8/8]	89.00 (64.00–222.00) [6/8]	209.00 (119.00–316.00) [5/8]	166.00 (74.50–325.00) [8/8]
Number of intromissions until the first ejaculation	18.50 (13.00–24.25) [8/8]	15.00 (13.75–18.00) [5/6]	21.50 (21.00–24.00) [4/5]	16.00 (13.50–17.00) [8/8]
Latency to the first ejaculation (s)	739.00 (619.50–944.50) [8/8]	616.00 (528.00–689.25) [5/6]	1157.00 (992.25–1284.75) [4/5]	911.00 (722.50–1231.50) [8/8]
Latency of the first post-ejaculatory intromission (s)	335.50 (310.25–356.25) [8/8]	311.10 (275.25–325.25) [5/6]	319.50 (270.00–361.50) [4/5]	280.00 (270.00–324.50) [8/8]
Number of post-ejaculatory intromissions	19.50 (14.00–26.00) [8/8]	20.00 (16.00–27.75) [5/6]	13.00 (11.75–15.50) [4/5]	13.00 (7.00–22.00) [8/8]
Number of ejaculations	2.00 (0.00–2.00) [8/8]	1.50 (1.00–1.50) [5/6]	2.00 (1.00–2.50) [4/5]	1.50 (1.00–2.00) [8/8]
Sexual incentive motivation	CTR [10]	TCS 0.8 [8]	TCS 2.4 [8]	TCS 8.0 [8]
Time spent in male zone (s)	278.97 ± 43.92	232.16 ± 23.92	304.56 ± 91.56	298.24 ± 39.67
Time spent in female zone (s)	618.03 ± 49.63	591.74 ± 51.00	583.35 ± 94.38	550.91 ± 47.76
Number of visits in male zone	17.40 ± 1.72	16.75 ± 0.53	13.88 ± 1.62	17.50 ± 1.36
Number of visits in female zone	20.60 ± 2.40	19.50 ± 1.31	17.00 ± 2.56	20.63 ± 1.86
Preference Score	68.69 ± 4.92	71.22 ± 3.26	65.76 ± 9.54	64.64 ± 4.88

All parameters of copulatory behavior are presented as median (1st and 3rd quartile) and were analyzed by the non-parametric test of Kruskal–Wallis, p > 0.05. Data on sexual incentive motivation are means ± SEM and were analyzed by ANOVA, p > 0.05. Numbers in brackets represent the number of animals per group that displayed the behavior. PND, postnatal day; CTR, corn oil; TCS 0.8, triclosan 0.8 mg/kg; TCS 2.4, triclosan 2.4 mg/kg; TCS 8.0, triclosan 8.0 mg/kg.

#### 3.1.3 Body Weight, AGD, Organ Weight, and Plasmatic Testosterone Quantification

The final body weight (g) at PND 140, AGD, organ weights, and plasma testosterone quantification of adult males of the F1 generation are presented in **Table 2**. No significant differences as a result of the TCS treatment were found in final body weight [F_(3,33)_ = 0.183, p = 0.907], AGD, [F_(3,33)_ = 0.488, p = 0.693], organ weights (ANOVA, p > 0.05), or plasma testosterone quantification (ng/dl) [F_(3,33)_ = 1.906, p = 0.150].

**Table 2:** Body weight, AGD, organ weights, and plasma testosterone quantification of male rats of F1 generation at PND 140.

	CTR [10]	TCS 0.8 [8]	TCS 2.4 [8]	TCS 8.0 [8]
Final body weight (g)	438.05 ± 17.43	451.81 ± 18.87	440.15 ± 24.54	432.43 ± 10.89
AGD (mm/g^1/3^)	5.09 ± 0.13	5.14 ± 0.06	4.97 ± 0.12	5.12 ± 0.09
Tests (g)	3.33 ± 0.10	3.25 ± 0.10	3.06 ± 0.11	3.38 ± 0.14
Vas deferens (left) (mg)	105.41 ± 4.28	113.31 ± 4.60	109.74 ± 4.17	114.86 ± 3.55
Epididymis (left) (mg)	613.01 ± 13.71	627.58 ± 17.32	581.20 ± 28.38	627.33 ± 17.63
Ventral prostate (g)	0.43 ± 0.04	0.49 ± 0.04	0.39 ± 0.04	0.39 ± 0.02
Liver (g)	14.42 ± 0.82	14.69 ± 0.75	14.58 ± 0.68	14.32 ± 0.57
Kidneys (g)	2.62 ± 0.11	2.65 ± 0.10	2.66 ± 0.10	2.69 ± 0.08
Adrenal glands (mg)	51.97 ± 2.92	53.03 ± 1.62	47.80 ± 2.36	51.51 ± 1.69
Seminal vesicle (g)	1.64 ± 0.08	1.62 ± 0.04	1.65 ± 0.12	1.58 ± 0.08
Testosterone (ng/dl)	192.12 ± 32.52	311.23 ± 38.81	234.99 ± 35.39	279.61 ± 48.12

Data are means ± SEM. Final body weight was compared using ANOVA (p > 0.05). Organ weight was considered through analysis of covariance (ANCOVA) on final body weight (p > 0.05). Numbers in brackets represent the number of animals per group. AGD, Anogenital distance; PND, postnatal day; CTR, corn oil; TCS 0.8, triclosan 0.8 mg/kg; TCS 2.4, triclosan 2.4 mg/kg; TCS 8.0, triclosan 8.0 mg/kg.

#### 3.1.4 Sperm Motility, Viability, Morphology, and Sperm Count

TCS exposure in F1 males affected the sperm viability, reducing the percentage of live sperm [F_(3.33)_ = 5.935, p = 0.003] and the percentage of mobile sperm [F_(3.33)_ = 4.666, p = 0.009] in males of the TCS 2.4 group, compared to the control animals (**Table 3**). No statistical differences were observed in the sperm count parameters, as shown in **Table 4** (p > 0.05).

**Table 3:** Sperm motility, viability, sperm count in the vas deferens, and morphology of male rats of F1 generation at PND 140.

	CTR [10]	TCS 0.8 [8]	TCS 2.4 [8]	TCS 8.0 [8]
Mobile sperm (%)	80.00 ± 2.34	80.63 ± 1.57	69.25 ± 2.72*	81.00 ± 3.34
Immobile sperm (%)	20.00 ± 2.34	19.38 ± 1.57	30.75 ± 2.72*	19.00 ± 3.34
Viable sperm (%)	81.50 ± 2.05	82.63 ± 1.24	70.88 ± 3.10*	83.63 ± 2.76
Non-viable sperm (%)	18.50 ± 2.05	17.38 ± 1.24	29.13 ± 3.10*	16.38 ± 2.76
Sperm count in the vas deferens (10^6^/ml)	55.85 ± 7.89	42.34 ± 4.02	39.59 ± 9.32	61.22 ± 13.75
Abnormal head morphology sperm (%)	18.96 ± 2.97	14.14 ± 2.96	16.69 ± 2.82	16.87 ± 2.21
Abnormal tail morphology sperm (%)	4.44 ± 0.79	2.17 ± 0.53	3.61 ± 0.74	2.55 ± 0.48

Data are means ± SEM. *p < 0.05 (ANOVA complemented with Bonferroni). Numbers in brackets represent the number of animals per group. PND, postnatal day; CTR, corn oil; TCS 0.8, triclosan 0.8 mg/kg; TCS 2.4, triclosan 2.4 mg/kg; TCS 8.0, triclosan 8.0 mg/kg.

**Table 4:** Sperm count in the testis and epididymis of male rats of F1 generation at PND 140.

	CTR [10]	TCS 0.8 [8]	TCS 2.4 [8]	TCS 8.0 [8]
N° of spermatids (10^6^/testis)	87.63 ± 18.82	72.01 ± 11.66	84.90 ± 15.95	85.27 ± 11.57
N° of spermatids (10^6^/g/testis)	60.31 ± 11.62	52.01 ± 8.20	59.04 ± 10.95	57.58 ± 8.02
DSP (10^6^)	14.37 ± 3.09	11.80 ± 1.91	13.92 ± 2.61	13.98 ± 1.90
N° of sperm × 10^6^/caput + corpus of epididymis	91.14 ± 7.34	88.47 ± 3.50	102.48 ± 12.58	93.39 ± 8.61
N° of sperm × 10^6^/g/caput + corpus of epididymis	298.57 ± 19.27	291.63 ± 16.86	350.44 ± 31.46	301.09 ± 27.10
N° of sperm × 10^6^/cauda of epididymis	172.58 ± 13.69	168.54 ± 17.01	143.91 ± 25.36	192.93 ± 13.13
N° of sperm × 10^6^/g/cauda of epididymis	654.89 ± 42.29	642.47 ± 51.75	579.15 ± 76.39	713.72 ± 45.96
Sperm transit time through caput/corpus of epididymis (days)	8.53 ± 1.37	9.60 ± 2.23	11.99 ± 5.11	7.61 ± 1.16
Sperm transit time through cauda of epididymis (days)	16.64 ± 2.68	18.48 ± 4.54	14.37 ± 3.66	16.29 ± 3.01

Data are means ± SEM. ANOVA, p > 0.05. Numbers in brackets represent the number of animals per group. PND, postnatal day; DSP, daily sperm production; CTR, corn oil; TCS 0.8, triclosan 0.8 mg/kg; TCS 2.4, triclosan 2.4 mg/kg; TCS 8.0, triclosan 8.0 mg/kg.

#### 3.1.5 Testicular Histomorphometry

**Figure 2:**
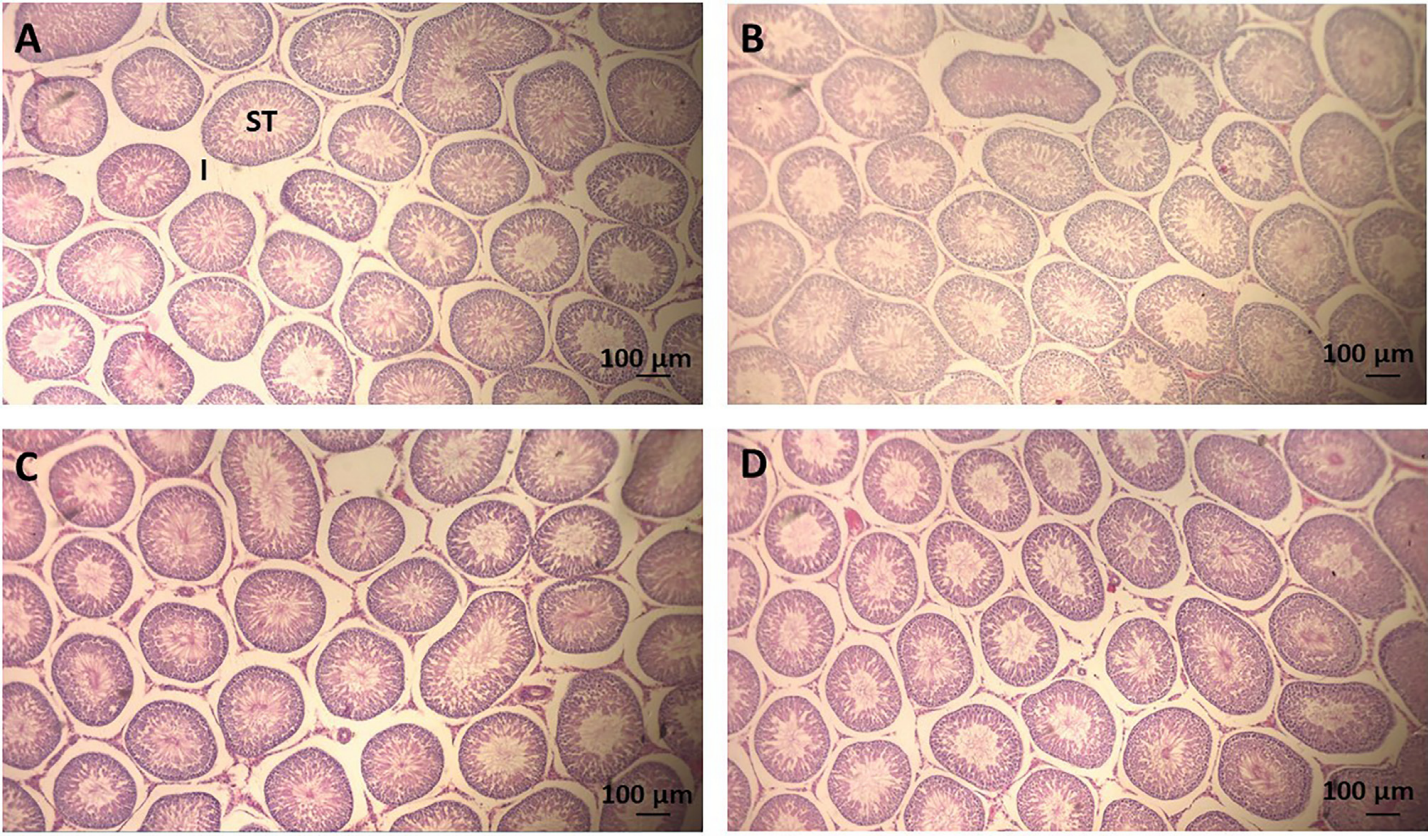
Photomicrography of sections of seminiferous tubules (ST) of adult rats (PND 140) of the F1 generation. Scale = 100 μm, hematoxylin-eosin stain. **(A)** CTR group, treated with corn oil; **(B)** TCS 0.8 group, treated with triclosan at a dose of 0.8 mg/kg; **(C)** TCS 2.4 group, treated with triclosan at a dose of 2.4 mg/kg; and **(D)** TCS 8.0 group, treated with triclosan at a dose of 8.0 mg/kg. ST, seminiferous tubule; I, Interstice.

The testicular histomorphometry results of the F1 generation are given in **Table 5**. No changes in testicular volume [F_(3,33)_ = 0.979, p = 0.416], interstitial content volume [F_(3,33)_ = 1.409, p = 0.259], seminiferous tubules volume [F_(3,33)_ = 0.513, p = 0.676], diameter of seminiferous tubules [F_(3,33)_ = 0.721, p = 0.547], and total length of seminiferous tubules [F_(3,33)_ = 0.276, p = 0.842] were detected by ANOVA complemented with Bonferroni. The testis parenchyma structure of rats on PND 140 of the F1 generation is shown in **Figure 2**.

**Table 5:** Volumetric composition (ml) and biometric evaluation of testicular parenchyma of male rats of F1 generation at PND 140.

	CTR [10]	TCS 0.8 [8]	TCS 2.4 [8]	TCS 8.0 [8]
Testicular volume (ml)	1.56 ± 0.05	1.52 ± 0.04	1.47 ± 0.05	1.59 ± 0.06
Volume of interstitial content (ml)	0.48 ± 0.03	0.48 ± 0.02	0.40 ± 0.03	0.47 ± 0.05
Volume of seminiferous tubules (ml)	1.08 ± 0.04	1.05 ± 0.04	1.07 ± 0.04	1.12 ± 0.03
Diameter of seminiferous tubules (µm)	285.87 ± 5.19	273.73 ± 9.85	278.57 ± 6.66	286.26 ± 6.63
Total length of seminiferous tubules (m)	16.97 ± 0.85	18.16 ± 1.35	17.64 ± 0.66	17.54 ± 0.83

Data are means ± SEM. ANOVA, p > 0.05. Numbers in brackets represent the number of animals per group. PND, postnatal day; CTR, corn oil; TCS 0.8, triclosan 0.8 mg/kg; TCS 2.4, triclosan 2.4 mg/kg; TCS 8.0, triclosan 8.0 mg/kg.

### 3.2 Parameters Analyzed During Development (F1 and F2 Generations)

#### 3.2.1 Body Weight

**Figure 3:**
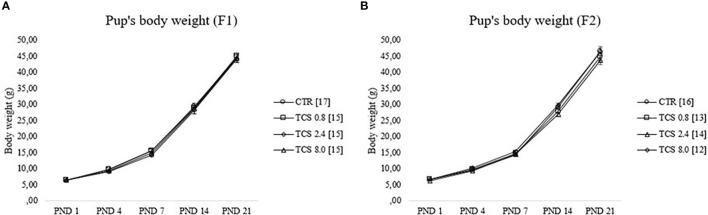
Pup’s body weight gain of male rats during lactational period [from postnatal day (PND) 1 until PND 21]. **(A, B)** represent data of F1 and F2 males, respectively. Data were reported as means ± SEM, n =12–17/group, p>0.05 (RMANOVA). CTR group, treated with corn oil; TCS 0.8 group, treated with triclosan at a dose of 0.8 mg/kg; TCS 2.4 group, treated with triclosan at a dose of 2.4 mg/kg; and TCS 8.0 group, treated with triclosan at a dose of 8.0 mg/kg.

Pup body weights (litter mean) of F1 and F2 generations during the first 3 weeks of life are illustrated in **Figure 3**. As expected, RMANOVA showed a significant (p < 0.05) effect of the age reflecting the weight gain of F1 and F2 pups throughout development; however, there was no interaction between age and treatment (p > 0.05), demonstrating a similar body weight gain among experimental groups.

#### 3.2.2 Sexual Development

The number of pups and sex ratio (M:F) were not affected by TCS treatment (data not shown). Relative AGD (mm/g1/3) of F1 and F2 male pups at PNDs 1, 4, and 21 were not influenced by TCS (p > 0.05) as indicated by RMANOVA. Nipple retention (% of males) on PND 12 was 100% in all experimental groups of both F1 and F2 generations (Chi-Square test, p > 0.05). ANOVA was performed to compare the age (day) in which preputial separation occurred in F1 males, and no difference was observed among the groups (p > 0.05) (data not shown).

#### 3.2.3 Physical and Neuromotor Development

Data from physical and neuromotor parameters of F1 and F2 pups are presented in **Table 6**. As indicated by the Kruskal-Wallis test, there were no significant differences in the days in which physical landmarks (incisor teeth eruption, ear opening, and eye opening) or ontogenic reflexes (surface righting reflex and negative geotaxis) occurred among experimental groups (p > 0.05).

**Table 6:** Physical and neuromotor development of male pups of F1 and F2 generations.

F1 generation	CTR [17]	TCS 0.8 [14]	TCS 2.4 [14]	TCS 8.0 [15]
Pinna detachment (day)	3.00 (3.00–3.00)	3.00 (3.00–3.00)	4.00 (3.00–4.00)	3.00 (3.00–4.00)
Surface righting reflex (day)	5.00 (5.00–5.00)	5.00 (5.00–5.00)	5.00 (5.00–5.00)	5.00 (5.00–5.00)
Incisor teeth eruption (day)	10.00 (9.00–11.00)	9.00 (9.00–9.75)	9.50 (9.00–10.00)	10.00 (9.00–10.00)
Negative geotaxis (day)	10.00 (9.00–10.00)	10.00 (9.00–11.00)	10.00 (9.25–10.75)	10.00 (9.00–10.50)
Eye opening (day)	14.00 (14.00–15.00)	14.00 (13.00–14.00)	14.00 (13.25–14.00)	14.00 (13.50–14.0)
F2 generation	CTR [14]	TCS 0.8 [12]	TCS 2.4 [12]	TCS 8.0 [11]
Pinna detachment (day)	3.00 (3.00–3.00)	3.00 (3.00–4.00)	3.00 (3.00–3.00)	3.00 (3.00–3.50)
Surface righting reflex (day)	5.00 (5.00–5.00)	5.00 (5.00–5.00)	5.00 (5.00–5.00)	5.00 (5.00–5.00)
Incisor teeth eruption (day)	9.00 (8.00–10.00)	9.00 (8.00–9.50)	9.00 (8.00–9.00)	9.00 (8.50–9.50)
Negative geotaxis (day)	9.00 (9.00–10.75)	10.00 (9.00–10.00)	9.00 (9.00–9.50)	10.00 (9.00–10.50)
Eye opening (day)	14.00 (14.00–14.00)	14.00 (14.00–15.00)	14.00 (14.00–15.00)	14.00 (13.50–14.00)

Data are medians (1st and 3rd quartiles). Numbers in brackets represent the number of animals per group. Kruskal–Wallis, p > 0.05. CTR, corn oil; TCS 0.8, triclosan 0.8 mg/kg; TCS 2.4, triclosan 2.4 mg/kg; TCS 8.0, triclosan 8.0 mg/kg.

#### 3.2.4 Motor Activity (Open Field)

Data from the behavioral evaluation of males from F1 and F2 generations in the open-field (locomotion and time spent in the central and peripheral areas) on PNDs 13, 17, and 21 are given in **Table 7**. RMANOVA showed a significant effect of age (p < 0.001) in the locomotor activity in both F1 and F2 groups, but without an age × treatment interaction (p > 0.05), indicating a similar increase in this behavior with aging for all the experimental groups. The total time spent in the central and peripheral areas were not statistically significant (p > 0.05).

**Table 7:** Locomotor behavior of male rats from F1 and F2 generations in the open-field test.

F1 generation	CTR [17]	TCS 0.8 [14]	TCS 2.4 [14]	TCS 8.0 [15]
PND 13				
Locomotion (n)	17.71 ± 3.32	22.21 ± 4.40	11.57 ± 3.23	27.87 ± 9.00
Time spent in central area (s)	88.96 ± 29.94	116.14 ± 24.30	148.03 ± 51.80	88.75 ± 21.70
Time spent in peripheral area (s)	511.04 ± 29.94	483.86 ± 24.30	451.97 ± 51.80	511.25 ± 21.70
PND 17				
Locomotion (n)	105.24 ± 19.25	111.14 ± 20.40	85.71 ± 15.17	113.27 ± 19.72
Time spent in central area (s)	95.26 ± 19.82	61.49 ± 11.71	91.31 ± 27.20	63.00 ± 14.28
Time spent in peripheral area (s)	504.74 ± 19.82	538.51 ± 11.71	508.69 ± 27.20	537.00 ± 14.28
PND 21				
Locomotion (n)	132.88 ± 8.52	149.14 ± 11.50	152.43 ± 13.26	162.67 ± 13.42
Time spent in central area (s)	165.73 ± 14.13	136.04 ± 17.76	146.16 ± 18.31	135.57 ± 13.57
Time spent in peripheral area (s)	434.27 ± 14.13	463.96 ± 17.76	453.84 ± 18.31	464.43 ± 13.57
F2 generation	CTR [14]	TCS 0.8 [12]	TCS 2.4 [12]	TCS 8.0 [11]
PND 13				
Locomotion (n)	34.21 ± 10.23	19.58 ± 3.05	23.17 ± 5.89	17.91 ± 7.30
Time spent in central area (s)	99.79 ± 27.87	107.50 ± 27.87	103.51 ± 17.88	105.54 ± 36.13
Time spent in peripheral area (s)	500.21 ± 27.87	492.50 ± 27.87	496.49 ± 17.88	494.46 ± 36.13
PND 17				
Locomotion (n)	154.00 ± 24.28	145.25 ± 25.95	150.50 ± 31.30	119.64 ± 19.38
Time spent in central area (s)	72.25 ± 15.15	67.63 ± 15.06	50.79 ± 11.52	46.56 ± 10.65
Time spent in peripheral area (s)	527.75 ± 15.15	532.37 ± 15.06	549.21 ± 11.52	553.44 ± 10.65
PND 21				
Locomotion (n)	164.36 ± 11.62	134.75 ± 9.55	135.50 ± 13.03	134.64 ± 12.50
Time spent in central area (s)	123.65 ± 12.33	89.59 ± 16.41	85.55 ± 11.74	87.65 ± 15.33
Time spent in peripheral area (s)	476.35 ± 12.33	510.41 ± 16.41	514.45 ± 11.74	512.35 ± 15.33

Data are means ± SEM. RMANOVA, p > 0.05. Numbers in brackets represent the number of animals per group. PND, postnatal day; CTR, corn oil; TCS 0.8, triclosan 0.8 mg/kg; TCS 2.4, triclosan 2.4 mg/kg; TCS 8.0, triclosan 8.0 mg/kg.

## 4 Discussion

Rodent and human exposure to TCS is frequently associated with decreased thyroxine levels with consequent impairments to reproductive and developmental health (58, 59). The purpose of this study was to investigate the reproductive and developmental effects of TCS in two generations of male rats throughout repeated oral treatment, germ cell, placenta, and lactation exposure. The doses used in this study were selected to determine the possible effects of low doses for risk assessment to the male reproductive system and were based on the acceptable daily intake of TCS allowed by the U. S. EPA (8), in addition to 3- and 10-fold higher doses.

In this study, male rats exposed to TCS throughout the intrauterine development and lactation until adulthood did not present alterations in body weight gain. This result is consistent with those observed in Wistar rats treated with TCS at doses ranging from 3 to 300 mg/kg (26, 28). Moreover, no signs of toxicity, such as abnormal respiratory pattern, lacrimation, piloerection, and tremors, were observed in either F1 or F2 males, suggesting that TCS treatment at the dose used does not cause general toxicity.

Regarding sexual behavior analysis of F1 male rats, no significant changes were observed in copulatory behavior or in sexual incentive motivation. These sexual behaviors are regulated by neurotransmitters and gonadal hormones, among them testosterone (60, 61). Some *in vitro* data indicate that TCS may exert adverse effects on reproductive functions. In rat testicular tissue the TCS exposure reduced the activity of steroidogenic enzymes: 3β- hydroxysteroid dehydrogenase and 17β- hydroxysteroid dehydrogenase (26), while in human embryonic kidney 293 cell, TCS inhibited transcriptional activity induced by testosterone (62), suggesting an antiandrogenic effect. In the present study, plasma testosterone levels and the weight of hormone-dependent organs at PND 140 have not been affected by low doses TCS exposure during intrauterine development, lactation, and subsequently, throughout development. Reduced plasma testosterone has been reported in 75-day-old F1 male offspring of Holtzman rats exposed to 0.1, 4, 40, and 150 mg/kg sc from GD 6 until LD 21 (29), as well as in pubertal (53 days old) Wistar rats gavaged with 200 mg/kg TCS for 31 days (28) and adult (130 days old) with 20 mg/kg TCS for 60 days (26). These latter studies also reported decreased weights of hormone-dependent organs, sperm production (26, 29), and motility (29).

A reduction in sperm production in male rats treated with TCS could be related to impairment involving hypothalamo-pituitary-gonadal axis by a decreased synthesis of LH and FSH (26). In Priyanka et al. (29), the TCS subcutaneous injections (0.1, 4, 40, and 150 mg/kg/day) from GD 6 to weaning (PND21) decreased expression of steroid hormone receptors (AR, ERa and ERb), StAR, and aromatase in F1 male rats, resulting in a dose-dependent decrease in testosterone level, sperm count, and motility. Even though testosterone and sperm production have not been affected in this study, we did observe reduced sperm motility and viability in the F1 generation exposed to 2.4 mg/kg at PND 140. Interestingly, the same low doses of TCS (0.8, 2.4, and 8.0 mg/kg, gavage) administered from PND 49 to PND 140 to Wistar rats did not affect the sperm parameters (36), indicating that TCS may be impacting the gametogenesis during fetal period. The reduction in sperm quality is commonly related to the toxic effects of a drug in the spermatogenesis process (63, 64) which is dependent on the action of testosterone (65). It is known that during fetal development, the gametogenesis is established, and any insult may affect the quality of gametes produced during this period (66). These results suggest that the effects of TCS on sperm quality may be influenced by the exposure window, since animals of the F1 generation were exposed to this compound throughout intrauterine development, lactation, and puberty, until adulthood. Evidence in mammals suggests that much of the programming of endocrine systems is consolidated during fetal/neonatal development (67) and that exposure to xenobiotics in these periods can permanently compromise the proper functioning of these systems (68, 69). Regarding the absence of effects at the lowest and highest doses (i.e., 0.8 and 8.0 mg/kg/day), it is known that the dose-response relationship for some EDs, including TCS, may depart from linearity at low oral doses. The occurrence of effects on sperm quality at the intermediate dose (2.4 mg/kg/day) suggests a non-monotonic dose-response effect for TCS (70, 71).

Despite the changes observed in the sperm parameters, in the current study, there was no effect of the chronic treatment with TCS on the volumetric composition, or on any of the biometric parameters (diameter of the seminiferous tubules and total length of the seminiferous tubules) of the testicular parenchyma of adult rats of the F1 generation. In Priyanka et al. (29), maternal exposure to TCS at 150 mg/kg sc from GD 6 to LD 21 induced a significant decrease in the number of stage VII–VIII seminiferous tubules in the testis of male rats of F1 offspring compared to the vehicle group and to the lowest dose 0.1 mg/kg group. The absence of changes in testicular biometry allows us to infer that the effects of TCS on the observed sperm parameters may be related to the direct action of the antibacterial on the epididymis. It is important to clarify that we did not perform the histopathological evaluation of the epididymis, once only the left epididymis was preserved for analysis of sperm count. The epididymis has a fundamental role in the functional transformation of sperm and its storage in a viable and ready for ejaculation state (72). In Sprague-Dawley rats (PND 42), after the administration of a single dose of TCS (50 mg/kg, *via* gavage), it was observed that, in the epididymis, the TCS has a longer retention time and a longer elimination half-life compared to testis and prostate plasma, showing a tendency for the accumulation of this compound in the epididymis and consequent histopathological damage (73). Furthermore, as described above, maternal exposure to TCS from GD 6 to LD 21 at 4, 40, and 150 mg/kg sc induced a significant decrease in sperm count per cauda of the epididymis in rats of F1 male offspring (29), reinforcing this hypothesis. Indeed, the absence of histopathological analysis of the epididymis restricts the discussion regarding the potential TCS effects on spermatic process. This lack of information in the literature reinforces the importance of future studies to provide a better understanding of the TCS effects on this target organ.

Regarding sexual development, exposure to TCS during intrauterine development, lactation, and, subsequently, throughout development did not alter the AGD of F1 and F2 males, which is a marker of fetal androgen action (74), and also did not affect the day of preputial separation (an indicator of puberty onset) of F1 males. These data reinforce the lack of effect of the exposure regimen adopted in this study on testosterone levels at these development windows, since both parameters are androgen-dependent (75).

In this study, TCS exposure did not induce significant alterations in physical, neuromotor, and motor activity development, in both F1 and F2 generations. Neuromotor evaluation (open field) during the lactation period allows identification of possible neurobehavioral alterations in the early life of these animals. These evaluations are important because the development of the offspring’s nervous system might be indirectly affected, since environmental toxicants are capable of inducing non-specific effects that include alterations in hormone release and/or the nutritional status of dams (76). These data show that TCS at the doses used did not influence the development of the nervous system of the male rats.

In summary, the treatment and maternal (placenta and lactation) exposure to TCS compromised sperm parameters in rats of the F1 generation, observed by a decrease in motility and viability of sperm at 2.4 mg/kg. This is the first study to examine the long-time effects of exposure to TCS through germ cells, placenta, lactation, and oral treatment on reproductive potential, including sexual behavior, and physical and neuromotor development in male Wistar rats. These results emphasize the importance of studying the safety of exposure to TCS on reproductive function for the population. Although the TCS toxic effects on sperm quality only occurred at the intermediate dose (2.4 mg/kg), it is important to clarify that the doses used in this study were based on the maximum limits of exposure to TCS in the diet recommended by regulatory agencies. As previously stated, the TCS 2.4 group received a higher dose (compared to TCS 0.8 group) to compensate for potential intraspecies variations, and these animals were susceptible to TCS sperm toxicity. A further epigenetic investigation is needed to clarify how exposure to TCS during important development windows affects the sperm quality of rats.

## Data Availability Statement

The original contributions presented in the study are included in the article/**Supplementary Material**. Further inquiries can be directed to the corresponding author.

## Ethics Statement

The animal study was reviewed and approved by the State University of Londrina Ethics Committee for Animal Research (CEUA/UEL: 283.2015.27).

## Author Contributions

BM conceived and designed the study. SF helped with data obtention and played an active role during manuscript drafting. KP and MM performed experiments of F1 and F2 generations. MP performed histological analysis. NC performed sperm parameters evaluation. EM contributed with the technical and data review. JA-F quantified plasmatic testosterone, and DG supervised and coordinated the study. All authors listed have contributed to the work and approved the final version.

## Funding

This work was supported by CAPES (Doctoral fellowship to BM) and CNPq (454499/2014-0).

## Conflict of Interest

The authors declare that the research was conducted in the absence of any commercial or financial relationships that could be construed as a potential conflict of interest.

## Publisher’s Note

All claims expressed in this article are solely those of the authors and do not necessarily represent those of their affiliated organizations, or those of the publisher, the editors and the reviewers. Any product that may be evaluated in this article, or claim that may be made by its manufacturer, is not guaranteed or endorsed by the publisher.

## References

[1] International Program on Chemical Safety (IPCS). Global Assessment of the State-of-the-Science of Endocrine Disruptors. WHO/PCS/EDC/02.2. Geneva: World Health Organization (2002). Available at: https://apps.who.int/iris/handle/10665/67357.

[2] U. S. Environmental Protection Agency (EPA). Special Report on Environmental Endocrine Disruption: An Effects Assessment and Analysis. Washington: EPA/630/R-96/012 (1997). Available at: https://nepis.epa.gov/Exe/ZyPDF.cgi/30004ZD3.PDF?Dockey=30004ZD3.PDF.

[3] Jurewicz J Radwan M Wielgomas B Kałużny P Klimowska A Radwan P. Environmental Levels of Triclosan and Male Fertility. Environ. Sci Pollut Res (2018) 25(6):5484–90. doi: 10.1007/s11356-017-0866-5 PMC5823964 29214481

[4] Schultz MM Bartell SE Schoenfuss HL. Effects of Triclosan and Triclocarban, Two Ubiquitous Environmental Contaminants, on Anatomy, Physiology, and Behavior of the Fathead Minnow (Pimephales Promelas). Arch Environ Contam Toxicol (2012) 63(1):114–24. doi: 10.1007/s00244-011-9748-x 22237462

[5] Ullah A Pirzada M Jahan S Ullah H Shaheen G Rehman H. Bisphenol A and its Analogs Bisphenol B, Bisphenol F, and Bisphenol S: Comparative *In Vitro* and *In Vivo* Studies on the Sperms and Testicular Tissues of Rats. ChemosphereV (2018) 209:508–16. doi: 10.1016/j.chemosphere.2018.06.089 29940534

[6] Bhargava HN Leonard PA. Triclosan: Applications and Safety. Am J Infect Control (2014) 24(3):209–18. doi: 10.1016/s0196-6553(96)90017-6 8807001

[7] United Nations Environment Programme (UNEP) World Health Organization (WHO). State-of-the-Science of Endocrine Disrupting Chemicals - 2012. Geneva: World Health Organization, World Health Organization (2013). Available at: https://apps.who.int/iris/bitstream/handle/10665/78102/WHO_HSE_PHE_IHE_2013.1_eng.pdf.

[8] U. S. Environmental Protection Agency. Reregistration Eligibility Decision for Triclosan: List B. Washington: EPA 739/RO/8009 (2008). Available at: https://www3.epa.gov/pesticides/chem_search/reg_actions/reregistration/red_PC-054901_18-Sep-08.pdf.

[9] Crofton KM Paul KB DeVito MJ Hedge JM. Short-Term *In Vivo* Exposure to the Water Contaminant Triclosan: Evidence for Disruption of Thyroxine. Environ Toxicol Pharmacol (2007) 24(2):194–7. doi: 10.1016/j.etap.2007.04.008 21783810

[10] Louis GW Hallinger DR Stoker TE. The Effect of Triclosan on the Uterotrophic Response to Extended Doses of Ethinyl Estradiol in the Weanling Rat. Reprod Toxicol (2013) 36:71–7. doi: 10.1016/j.reprotox.2012.12.001 23261820

[11] Stoker TE Gibson EK Zorrilla LM. Triclosan Exposure Modulates Estrogen-Dependent Responses in the Female Wistar Rat. Toxicol Sci (2010) 117(1):45–53. doi: 10.1093/toxsci/kfq180 20562219

[12] Veldhoen N Skirrow RC Osachoff H Wigmore H Clapson DJ Gunderson MP. The Bactericidal Agent Triclosan Modulates Thyroid Hormone-Associated Gene Expression and Disrupts Postembryonic Anuran Development. Aquat Toxicol (2006) 80(3):217–27. doi: 10.1016/j.aquatox.2006.08.010 17011055

[13] EPA-Denmark. Survey of Triclosan in Cosmetic Products No. 152. København: Environmental Protection Agency (2016). Available at: https://www2.mst.dk/Udgiv/publications/2016/12/978-87-93529-47-2.pdf.

[14] Bedoux G Roig B Thomas O Dupont V Le Bot B. Occurrence and Toxicity of Antimicrobial Triclosan and by-Products in the Environment. Environ Sci Pollut Res (2012) 19(4):1044–65. doi: 10.1007/s11356-011-0632-z 22057832

[15] Chu S Metcalfe CD. Simultaneous Determination of Triclocarban and Triclosan in Municipal Biosolids by Liquid Chromatography Tandem Mass Spectrometry. J Chromatogr A (2007) 1164(1–2):212–8. doi: 10.1016/j.chroma.2007.07.024 17692856

[16] Yueh M-F Tukey RH. Triclosan: A Widespread Environmental Toxicant With Many Biological Effects. Annu Rev Pharmacol Toxicol (2016) 56(1):251–72. doi: 10.1146/annurev-pharmtox-010715-103417 PMC4774862 26738475

[17] Mortensen ME Calafat AM Ye X Wong L-Y Wright DJ Pirkle JL. Urinary Concentrations of Environmental Phenols in Pregnant Women in a Pilot Study of the National Children’s Study. Environ Res (2014) 129:32–8. doi: 10.1016/j.envres.2013.12.004 PMC4530794 24529000

[18] Allmyr M Adolfsson-Erici M McLachlan MS Sandborgh-Englund G. Triclosan in Plasma and Milk From Swedish Nursing Mothers and Their Exposure via Personal Care Products. Sci Total Environ (2006) 372(1):87–93. doi: 10.1016/j.scitotenv.2006.08.007 17007908

[19] Jackson EN Rowland-Faux L James MO Wood CE. Administration of Low Dose Triclosan to Pregnant Ewes Results in Placental Uptake and Reduced Estradiol Sulfotransferase Activity in Fetal Liver and Placenta. Toxicol Lett (2018) 294:116–21. doi: 10.1016/j.toxlet.2018.05.014 PMC6026481 29772265

[20] Allmyr M Harden F Toms L-ML Mueller JF McLachlan MS Adolfsson-Erici M. The Influence of Age and Gender on Triclosan Concentrations in Australian Human Blood Serum. Sci Total Environ (2008) 393(1):162–7. doi: 10.1016/j.scitotenv.2007.12.006 18207219

[21] Perez AL De Sylor MA Slocombe AJ Lew MG Unice KM Donovan EP. Triclosan Occurrence in Freshwater Systems in the United States (1999-2012): A Meta-Analysis. Environ Toxicol Chem (2013) 32(7):1479–87. doi: 10.1002/etc.2217 23471841

[22] Wang C-F Tian Y. Reproductive Endocrine-Disrupting Effects of Triclosan: Population Exposure, Present Evidence and Potential Mechanisms. Environ Pollut (2015) 206:195–201. doi: 10.1016/j.envpol.2015.07.001 26184583

[23] Giuliano CA Rybak MJ. Efficacy of Triclosan as an Antimicrobial Hand Soap and Its Potential Impact on Antimicrobial Resistance: A Focused Review. Pharmacother J Hum Pharmacol Drug Ther (2015) 35(3):328–36. doi: 10.1002/phar.1553 25809180

[24] Food and Drug Administration H. Safety and Effectiveness of Consumer Antiseptics; Topical Antimicrobial Drug Products for Over-the-Counter Human Use. Final Rule. Fed. Regist (2016) 81:172. 27632802

[25] Kumar V Balomajumder C Roy P. Disruption of LH-Induced Testosterone Biosynthesis in Testicular Leydig Cells by Triclosan: Probable Mechanism of Action. Toxicology (2008) 250(2–3):124–31. doi: 10.1016/j.tox.2008.06.012 18655822

[26] Kumar V Chakraborty A Kural MR Roy P. Alteration of Testicular Steroidogenesis and Histopathology of Reproductive System in Male Rats Treated With Triclosan. Reprod Toxicol (2009) 27(2):177–85. doi: 10.1016/j.reprotox.2008.12.002 19118620

[27] Zhu W Zhang H Tong C Xie C Fan G Zhao S. Environmental Exposure to Triclosan and Semen Quality. Int J Environ Res Public Health (2016) 13(2):224. doi: 10.3390/ijerph13020224 26901211 PMC4772244

[28] Zorrilla LM Gibson EK Jeffay SC Crofton KM Setzer WR Cooper RL. The Effects of Triclosan on Puberty and Thyroid Hormones in Male Wistar Rats. Toxicol Sci (2009) 107(1):56–64. doi: 10.1093/toxsci/kfn225 18940961

[29] Priyanka Trivedi A Maske P Mote C Dighe V. Gestational and Lactational Exposure to Triclosan Causes Impaired Fertility of F1 Male Offspring and Developmental Defects in F2 Generation. Environ Pollut (2020) 257:113617. doi: 10.1016/j.envpol.2019.113617 31780364

[30] Wang X Chen X Feng X Chang F Chen M Xia Y. Triclosan Causes Spontaneous Abortion Accompanied by Decline of Estrogen Sulfotransferase Activity in Humans and Mice. Sci Rep (2015) 5(1):18252. doi: 10.1038/srep18252 26666354 PMC4678904

[31] Muth-Köhne E Wichmann A Delov V Fenske M. The Classification of Motor Neuron Defects in the Zebrafish Embryo Toxicity Test (ZFET) as an Animal Alternative Approach to Assess Developmental Neurotoxicity. Neurotoxicol Teratol (2012) 34(4):413–24. doi: 10.1016/j.ntt.2012.04.006 22729072

[32] Gillman MW. Developmental Origins of Health and Disease. N Engl J Med (2005) 353(17):1848–50. doi: 10.1056/NEJMe058187 PMC1488726 16251542

[33] Suzuki K. The Developing World of DOHaD. J Dev Orig Health Dis (2018) 9(3):266–9. doi: 10.1017/S2040174417000691 28870276

[34] OECD/OCDE. Test No. 416: Two-Generation Reproduction Toxicity. OECD Guidelines for the Testing of Chemicals, Section 4. Paris: OECD Publishing (2001). doi: 10.1787/9789264070868-en

[35] OECD/OCDE. Test No. 426: Developmental Neurotoxicity Study. OECD Guidelines for the Testing of Chemicals. Paris: OECD Publishing (2007). doi: 10.1787/9789264067394-en

[36] Pernoncini KV Montagnini BG de Góes MLM Garcia PC Gerardin DCC. Evaluation of Reproductive Toxicity in Rats Treated With Triclosan. Reprod Toxicol (2018) 75:65–72. doi: 10.1016/j.reprotox.2017.11.010 29197580

[37] Umbuzeiro GA. Guia De Potabilidade Para Substâncias Químicas. São Paulo: Associação Brasileira de Engenharia Sanitária e Ambiental (2012). Available at: https://www.abas.org/arquivos/guiapotabilidade.pdf.

[38] World Health Organization (WHO). Guidelines for Drinking-Water Quality. 4th ed. Geneva: World Health Organization (2011). Available at: https://www.who.int/publications/i/item/9789241549950.

[39] U. S. Environmental Protection Agency (EPA) Office of the Science Advisor Risk Assessment Forum. Recommended Use of Body Weight 3/4 as the Default Method in Derivation of the Oral Reference Dose. Washington: EPA/100/R11/0001 (2011). Available at: https://www.epa.gov/sites/default/files/2013-09/documents/recommended-use-of-bw34.pdf.

[40] Montagnini BG Pernoncine KV Borges LI Costa NO Moreira EG Anselmo-Franci JA. Investigation of the Potential Effects of Triclosan as an Endocrine Disruptor in Female Rats: Uterotrophic Assay and Two-Generation Study. Toxicology (2018) 410:152–65. doi: 10.1016/j.tox.2018.10.005 30321646

[41] Gerardin DCC Bernardi MM Moreira EG Pereira OCM. Neuroendocrine and Reproductive Aspects of Adult Male Rats Exposed Neonatally to an Antiestrogen. Pharmacol Biochem Behav (2006) 83(4):618–23. doi: 10.1016/j.pbb.2006.03.026 16650888

[42] Ågmo A. Male Rat Sexual Behavior. Brain Res Protoc (1997) 1(2):203–9. doi: 10.1016/S1385-299X(96)00036-0 9385085

[43] Ågmo A. Lack of Opioid or Dopaminergic Effects on Unconditioned Sexual Incentive Motivation in Male Rats. Behav Neurosci (2003) 117(1):55–98. doi: 10.1037/0735-7044.117.1.55 12619908

[44] Perobelli JE Martinez MF da Silva Franchi CA Fernandez CDB Camargo JLV Kempinas WDG. Decreased Sperm Motility in Rats Orally Exposed to Single or Mixed Pesticides. J Toxicol Environ Heal Part A (2010) 73(13–14):991–1002. doi: 10.1080/15287391003751802 20563933

[45] Favareto APA Fernandez CDB da Silva DAF Anselmo-Franci JA Kempinas WDG. Persistent Impairment of Testicular Histology and Sperm Motility in Adult Rats Treated With Cisplatin at Peri-Puberty. Basic Clin Pharmacol Toxicol (2011) 109(2):85–96. doi: 10.1111/j.1742-7843.2011.00688.x 21410649

[46] Bjorndahl L. Evaluation of the One-Step Eosin-Nigrosin Staining Technique for Human Sperm Vitality Assessment. Hum Reprod (2003) 18(4):813–6. doi: 10.1093/humrep/deg199 12660276

[47] Piffer RC Garcia PC Gerardin DCC Kempinas WG Pereira OCM. Semen Parameters, Fertility and Testosterone Levels in Male Rats Exposed Prenatally to Betamethasone. Reprod Fertil Dev (2009) 21(5):634–9. doi: 10.1071/RD08203 19486599

[48] Fernandes GSA Arena AC Fernandez CDB Mercadante A Barbisan LF Kempinas WG. Reproductive Effects in Male Rats Exposed to Diuron. Reprod Toxicol (2007) 23(1):106–12. doi: 10.1016/j.reprotox.2006.09.002 17070669

[49] Seed J Chapin RE Clegg ED Dostal LA Foote RH Hurtt ME. Methods for Assessing Sperm Motility, Morphology, and Counts in the Rat, Rabbit, and Dog: A Consensus Report. Reprod Toxicol (1996) 10(3):237–44. doi: 10.1016/0890-6238(96)00028-7 David. 8738562

[50] Filler R. Methods for Evaluation of Rat Epididymal Sperm Morphology. In: Chapin RE Heindel JH, editors. Methods in Toxicology: Male Reproductive Toxicology. San Diego, CA: Academic Press (1993). p. 334–43.

[51] Robb GW Amann RP Killian GJ. Daily Sperm Production and Epididymal Sperm Reserves of Pubertal and Adult Rats. Reproduction (1978) 54(1):103–7. doi: 10.1530/jrf.0.0540103 712697

[52] França LR Godinho CL. Testis Morphometry, Seminiferous Epithelium Cycle Length, and Daily Sperm Production in Domestic Cats (Felis Catus). Biol Reprod (2003) 68(5):1554–61. doi: 10.1095/biolreprod.102.010652 12606460

[53] Attal J Courot M Richetin C Pisselet C. Développement Testiculaire Et Établissement De La Spermatogenèse Chez Le Taureau. Ann Biol Anim Biochim Biophys (1963) 3(3):219–41. doi: 10.1051/rnd:19630302

[54] Dorst VJ Sajonski H. Morphometrische Untersuchunhen am Tubulussystem Des Schweinehodens Während Der Postnatalen Entwicklug. Monatshafte für Vet Medizine (1974) 29:650–2. 4449543

[55] Gallavan RH Holson JF Stump DG Knapp JF Reynolds VL. Interpreting the Toxicologic Significance of Alterations in Anogenital Distance: Potential for Confounding Effects of Progeny Body Weights. Reprod Toxicol (1999) 13(5):383–90. doi: 10.1016/S0890-6238(99)00036-2 10560587

[56] Heyser CJ. Assessment of Developmental Milestones in Rodents. Curr Protoc Neurosci (2003) 25(1):1–15. doi: 10.1002/0471142301.ns0818s25 18428605

[57] Moreira EG Vassilieff I Vassilieff VS. Developmental Lead Exposure: Behavioral Alterations in the Short and Long Term. Neurotoxicol Teratol (2001) 23(5):489–95. doi: 10.1016/S0892-0362(01)00159-3 11711252

[58] Johnson PI Koustas E Vesterinen HM Sutton P Atchley DS Kim AN. Application of the Navigation Guide Systematic Review Methodology to the Evidence for Developmental and Reproductive Toxicity of Triclosan. Environ Int (2016) 92–93:716–28. doi: 10.1016/j.envint.2016.03.009 PMC4951161 27156197

[59] Ruszkiewicz JA Li S Rodriguez MB Aschner M. Is Triclosan a Neurotoxic Agent? J Toxicol Environ Heal Part B (2017) 20(2):104–17. doi: 10.1080/10937404.2017.1281181 28339349

[60] Canteras NS. Hypothalamic Goal-Directed Behavior–Ingestive, Reproductive and Defensive. In: Watson C Paxinos G Puelles L, editors. The Mouse Nervous System. Elsevier Academic Press (2012). p. 539–62. doi: 10.1016/B978-0-12-369497-3.10020-2

[61] Hull EM Dominguez JM. Getting His Act Together: Roles of Glutamate, Nitric Oxide, and Dopamine in the Medial Preoptic Area. Brain Res (2006) 1126(1):66–75. doi: 10.1016/j.brainres.2006.08.031 16963001

[62] Chen J Ahn KC Gee NA Gee SJ Hammock BD Lasley BL. Antiandrogenic Properties of Parabens and Other Phenolic Containing Small Molecules in Personal Care Products. Toxicol Appl Pharmacol (2007) 221(3):278–84. doi: 10.1016/j.taap.2007.03.015 PMC1978490 17481686

[63] Ku WW Chapin RE Wine RN Gladen BC. Testicular Toxicity of Boric Acid (BA): Relationship of Dose to Lesion Development and Recovery in the F344 Rat. Reprod Toxicol (1993) 7(4):305–19. doi: 10.1016/0890-6238(93)90020-8 8400621

[64] Poon R. Short-Term Oral Toxicity of Pentyl Ether, 1,4-Diethoxybutane, and 1,6-Dimethoxyhexane in Male Rats. Toxicol Sci (2003) 77(1):142–50. doi: 10.1093/toxsci/kfg251 14657524

[65] Pakarainen T Zhang F-P Mäkelä S Poutanen M Huhtaniemi I. Testosterone Replacement Therapy Induces Spermatogenesis and Partially Restores Fertility in Luteinizing Hormone Receptor Knockout Mice. Endocrinology (2005) 146(2):596–606. doi: 10.1210/en.2004-0913 15514086

[66] Campagna C Sirard M-A Ayotte P Bailey JL. Impaired Maturation, Fertilization, and Embryonic Development of Porcine Oocytes Following Exposure to an Environmentally Relevant Organochlorine Mixture. Biol Reprod (2001) 65(2):554–60. doi: 10.1095/biolreprod65.2.554 11466225

[67] De Kloet ER Rosenfeld P Van Eekelen JAM Sutanto W Levine S. Stress, Glucocorticoids and Development. Prog Brain Res (1988) 73:101–20. doi: 10.1016/S0079-6123(08)60500-2 3047791

[68] Gutleb AC Cambier S Serchi T. Impact of Endocrine Disruptors on the Thyroid Hormone System. Horm Res Paediatr (2016) 86(4):271–8. doi: 10.1159/000443501 26771660

[69] Rock KD Patisaul HB. Environmental Mechanisms of Neurodevelopmental Toxicity. Curr Environ Heal Rep (2018) 5(1):145–57. doi: 10.1007/s40572-018-0185-0 PMC6377563 29536388

[70] Vandenberg LN Welshons WV vom Saal FS Toutain P Myers JP. Should Oral Gavage be Abandoned in Toxicity Testing of Endocrine Disruptors? Environ Heal (2014) 13(1):46. doi: 10.1186/1476-069X-13-46 PMC4069342 24961440

[71] Vandenberg LN Colborn T Hayes TB Heindel JJ Jacobs DR Lee D-H. Hormones and Endocrine-Disrupting Chemicals: Low-Dose Effects and Nonmonotonic Dose Responses. Endocr Rev (2012) 33(3):378–455. doi: 10.1210/er.2011-1050 22419778 PMC3365860

[72] Zhou W De Iuliis GN Dun MD Nixon B. Characteristics of the Epididymal Luminal Environment Responsible for Sperm Maturation and Storage. Front Endocrinol (2018) 9:59. doi: 10.3389/fendo.2018.00059 PMC5835514 29541061

[73] Lan Z Kim TH Bi KS Chen XH Kim HS. Triclosan Exhibits a Tendency to Accumulate in the Epididymis and Shows Sperm Toxicity in Male Sprague-Dawley Rats. Environ Toxicol (2015) 30(1):83–91. doi: 10.1002/tox.21897 23929691

[74] Welsh M Saunders PTK Fisken M Scott HM Hutchison GR Smith LB. Identification in Rats of a Programming Window for Reproductive Tract Masculinization, Disruption of Which Leads to Hypospadias and Cryptorchidism. J Clin Invest (2008) 118(4):1479–90. doi: 10.1172/JCI34241 PMC2267017 18340380

[75] Clark RL Antonello JM Grossman SJ Wise LD Anderson C Bagdon WJ. External Genitalia Abnormalities in Male Rats Exposed In Utero to Finasteride, a 5α-Reductase Inhibitor. Teratology (1990) 42(1):91–100. doi: 10.1002/tera.1420420111 2168096

[76] Spear LP. Neurobehavioral Assessment During the Early Postnatal Period. Neurotoxicol Teratol (1990) 12(5):489–95. doi: 10.1016/0892-0362(90)90012-2 2247037

