# Supplementary figures and images for: Developmental and Reproductive Outcomes in Male Rats Exposed to Triclosan: Two-Generation Study
Source: Front Endocrinol (Lausanne). 2021 Oct 13;12:738980. doi: 10.3389/fendo.2021.738980 (PMC8548666; doi:10.3389/fendo.2021.738980)

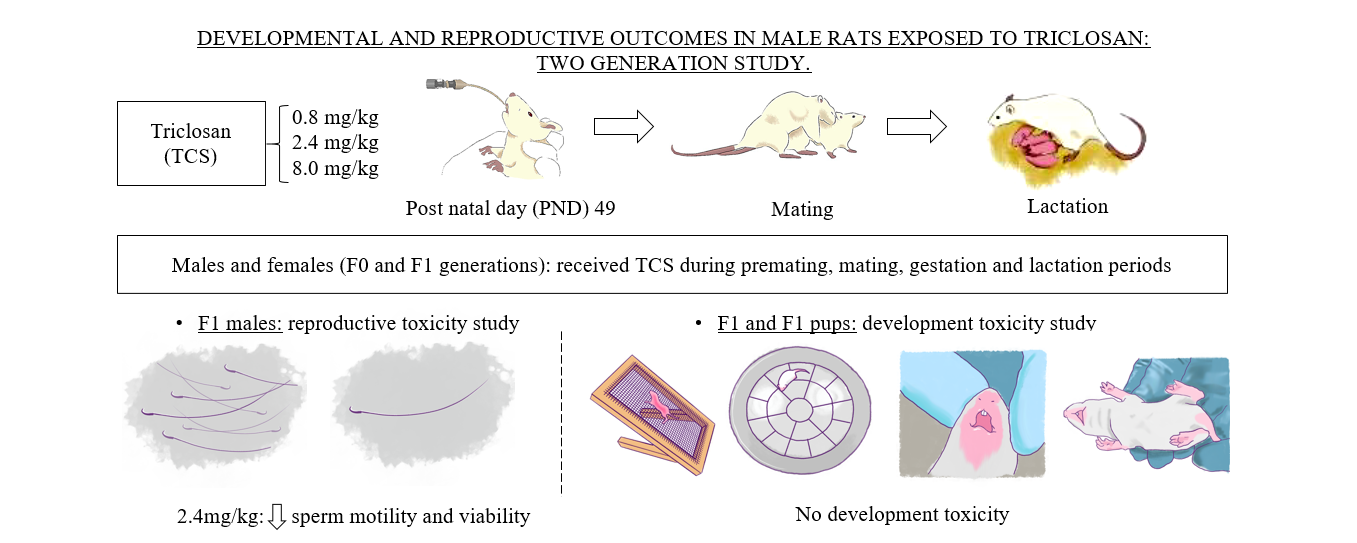

Supplement: Supplementary file 1 [file Image_1.tif]
